# Distinct T cell signatures are associated with *Staphylococcus* *aureus* skin infection in pediatric atopic dermatitis

**DOI:** 10.1172/jci.insight.178789

**Published:** 2024-05-08

**Authors:** Julianne Clowry, Daniel J. Dempsey, Tracey J. Claxton, Aisling M. Towell, Mary B. Turley, Martin Sutton, Joan A. Geoghegan, Sanja Kezic, Ivone Jakasa, Arthur White, Alan D. Irvine, Rachel M. McLoughlin

**Affiliations:** 1 Department of Dermatology, National Children’s Research Centre, Children’s Health Ireland at Crumlin, Dublin, Ireland.; 2 Clinical Medicine, Trinity College Dublin, Dublin, Ireland.; 3 Host-Pathogen Interactions Group, School of Biochemistry and Immunology, Trinity Biomedical Sciences Institute, Trinity College Dublin, Dublin, Ireland.; 4 Department of Microbiology, Moyne Institute of Preventive Medicine, School of Genetics and Microbiology, Trinity College Dublin, Dublin, Ireland.; 5 Institute of Microbiology and Infection, College of Medical and Dental Sciences, University of Birmingham, Birmingham, United Kingdom.; 6 Amsterdam UMC, University of Amsterdam, Department of Public and Occupational Health, Amsterdam Public Health Research Institute, Amsterdam, Netherlands.; 7 Laboratory for Analytical Chemistry, Department of Chemistry and Biochemistry, Faculty of Food Technology and Biotechnology, University of Zagreb, Zagreb, Croatia.; 8 School of Computer Science and Statistics, Trinity College Dublin, Dublin, Ireland.

**Keywords:** Dermatology, Immunology, Adaptive immunity

## Abstract

Atopic dermatitis (AD) is an inflammatory skin condition with a childhood prevalence of up to 25%. Microbial dysbiosis is characteristic of AD, with *Staphylococcus aureus* the most frequent pathogen associated with disease flares and increasingly implicated in disease pathogenesis. Therapeutics to mitigate the effects of *S*. *aureus* have had limited efficacy and *S*. *aureus*–associated temporal disease flares are synonymous with AD. An alternative approach is an anti–*S*. *aureus* vaccine, tailored to AD. Experimental vaccines have highlighted the importance of T cells in conferring protective anti–*S*. *aureus* responses; however, correlates of T cell immunity against *S*. *aureus* in AD have not been identified. We identify a systemic and cutaneous immunological signature associated with *S*. *aureus* skin infection (*AD_S.aureus_*) in a pediatric AD cohort, using a combined Bayesian multinomial analysis. *AD_S.aureus_* was most highly associated with elevated cutaneous chemokines IP10 and TARC, which preferentially direct Th1 and Th2 cells to skin. Systemic CD4^+^ and CD8^+^ T cells, except for Th2 cells, were suppressed in *AD_S.aureus_*, particularly circulating Th1, memory IL-10^+^ T cells, and skin-homing memory Th17 cells. Systemic γδ T cell expansion in *AD_S.aureus_* was also observed. This study suggests that augmentation of protective T cell subsets is a potential therapeutic strategy in the management of *S*. *aureus* in AD.

## Abstract 2

Characterization of immune signaling patterns in children with infected flares of eczema identifies potential therapeutic targets and strategies.

## Graphical abstract

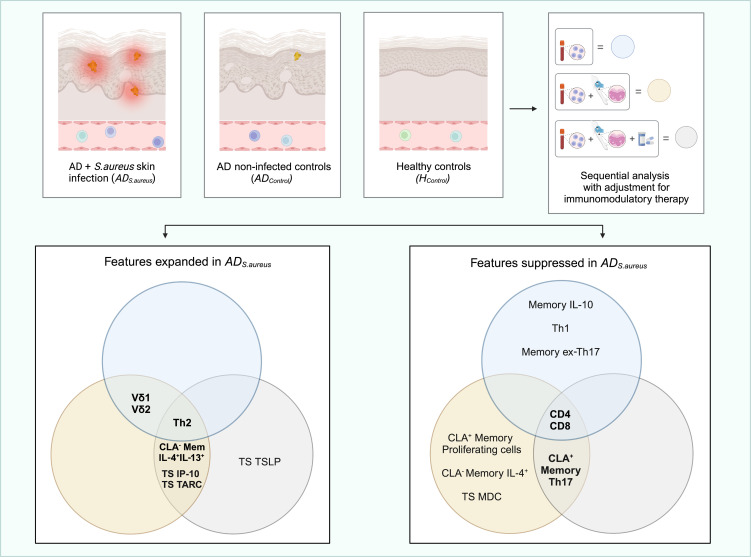

## Introduction

Atopic dermatitis (AD) is the most common chronic inflammatory skin disease of childhood (1), with a reported prevalence of up to 25% (2). Genetic and environmental factors, including microbial dysbiosis, are implicated in the epidermal barrier dysfunction and immune dysregulation that is characteristic of the condition. *Staphylococcus aureus* is the most frequent pathogen causing flares of AD (3). Dominant overgrowth of *S*. *aureus*, leading to a loss of skin microbial diversity, frequently precedes clinical deterioration in AD and may progress to overt infection (4). There is also evidence to suggest a causative role for this bacterium in disease pathogenesis. Epicutaneous exposure to *S*. *aureus* in a murine model is associated with the induction of IgE (5), while colonization with *S*. *aureus* in early life may also contribute to AD onset in infancy (6, 7). Patients with AD have consistently high levels of *S*. *aureus* colonization (8), with a pooled prevalence of 70% on lesional skin and 62% in the nose (9) compared with healthy control individuals who typically have much lower observed skin colonization rates of approximately 10% (10) and nasal colonization rates of approximately 20% (11). Recognized sequelae of *S*. *aureus* colonization in AD include greater type 2 immune deviation, increased barrier disruption, and more allergen sensitization compared with noncolonized AD individuals (12). In addition, *S*. *aureus* may attenuate protective host T cell immune responses (13, 14).

Therapeutic options to mitigate the effects of *S*. *aureus* in AD generally have had a limited and transient response. Treatment approaches in AD include barrier repair (15), decolonization (16), augmentation of skin commensals (17), antimicrobials and topical medications (18), or systemic immunomodulatory medications (19, 20). There is a declining role for antibiotic decolonization strategies in the context of rising antimicrobial resistance (21); however, short-term use of systemic antibiotics is still indicated in the setting of moderate-to-severe infection. Although the skin is the primary site for *S*. *aureus* infection in AD, rare, life-threatening systemic infections may arise, including bacteremia, septic shock, native-valve endocarditis, osteomyelitis, and necrotizing pneumonia (22).

Systemic immunomodulatory drugs are increasingly used in clinical practice to address the immune dysregulation predisposing to *S*. *aureus* colonization, infection, and increased AD disease activity (23). Th2 polarization is particularly relevant in the pathogenesis of AD and is associated with *S*. *aureus*–mediated disease flares (24). Th2 signaling promotes *S*. *aureus* hegemony and is amplified by the bacteria in a positive feedback loop (12). Drugs specifically targeting this pathway, such as dupilumab, an IL-4 receptor α antagonist and tralokinumab, a monoclonal antibody neutralizing IL-13, are associated with decreased *S*. *aureus* colonization and improved cutaneous microbial diversity (25, 26). Neither drug, however, fully eliminates *S*. *aureus* carriage. Despite clinical efficacy in a subgroup of AD patients, between 20% and 41% of patients, depending on treatment response criteria, are classified as nonresponders (27–29). This suggests that additional immune signaling pathways beyond Th2 must be targeted to eradicate *S*. *aureus* in AD (30).

Vaccination against *S*. *aureus* in AD is an attractive prospect, with the potential not only to limit the severity of AD but also to reduce the risk of severe complications and progression to other atopic diseases (31–33). Despite multiple preclinical and clinical trials over the last 20 years, a safe and clinically efficacious anti–*S*. *aureus* vaccine remains elusive. Limitations in previous vaccine studies range from inappropriate antigen selection to an excessive reliance on murine models that do not fully replicate in vivo human responses (34). Dependence on an opsonic antibody response, with the exclusion of cellular responses, has been a major factor in previous vaccine failures. Identifying correlates of cellular T cell immunity in high-risk patient cohorts is now considered an essential prerequisite for successful vaccine development (35). This is supported by evidence from human phase III studies of candidate anti–*S*. *aureus* vaccines, which failed despite generating robust humoral responses (36, 37), with post hoc analysis in one trial identifying undetectable IL-2 and IL-17A associated with *S*. *aureus* infections after vaccination (38). Murine models have also comprehensively shown that targeting Th1/Th17 cellular responses in conjunction with humoral immunity is effective in limiting *S*. *aureus* systemic and cutaneous infections (39, 40). No vaccine trials have focused on a human AD cohort, despite the disproportionate impact of *S*. *aureus* in this condition. As a result, correlates of a clinically protective T cell response to *S*. *aureus* in this population have not been described.

This study provides important insights into circulating *S*. *aureus* antigen–specific memory T cell responses, and local cutaneous cytokine signaling in *S*. *aureus*–infected AD (*AD_S.aureus_*), noninfected AD (*AD_control_*), and healthy controls (*H_control_*) using a combined Bayesian multinomial analysis, which identified a distinct immune signature associated with *AD_S.aureus_*. In *AD_S.aureus_*, cutaneous cytokines interferon-inducible protein 10 (IP10, also known as CXCL10) and thymus- and activation-regulated chemokine (TARC, also referred to as CCL17), which play a key role in Th1 and Th2 cutaneous trafficking, were elevated compared with *AD_control_* and *H_control_*. Systemic CD4^+^ and CD8^+^ T cell signaling, except for Th2 responses, was suppressed in *AD_S.aureus_* compared with the other groups, particularly circulating Th1 and memory IL-10^+^ T cells. Interestingly, an expansion of systemic γδ T cells was observed in *AD_S.aureus_*, suggesting a compensatory γδ T cell response in AD in the setting of conventional αβ T cell suppression. This study enhances our understanding of T cell immune responses to *S*. *aureus* skin infection in AD and provides insights into potential correlates of immunity, an essential prerequisite for anti–*S*. *aureus* vaccine design.

## Results

### Patient characteristics.

Ninety-three patients, ranging in age from 0 to 16 years, were recruited over a 10-month period from a single center. Twelve patients with AD were confirmed to have an *S*. *aureus* skin infection based on clinical criteria (3) that included weeping, pustule, abscess and or crust formation, and positive bacterial swab results (*AD_S.aureus_*). The remaining 46 patients with AD did not meet clinical criteria for diagnosis of an *S*. *aureus* skin infection (*AD_control_*). Patients were stratified based on clinical signs of *S*. *aureus* infection rather than colonization status, in order to identify features of an active immune response to infection as opposed to colonization, which likely involves a distinct immunological phenotype (12), given the heterogeneity in skin colonization status (low/medium/high) that can be challenging to determine clinically, and the fact that colonization may involve multiple sites, including the skin and nares, but also the gut (41). Additionally, defining groups based on colonization status at a single time point fails to identify transient or persistent colonization status. Thus, it is challenging in practice to definitively stratify patients based on colonization status.

There were 35 *H_control_* participants who did not have an active *S*. *aureus* skin infection or history of atopy.

Systemic immunological profiles were obtained from all 93 patients and a subset of 69 had systemic and local skin immune profiles completed. Within this subset of 69, there were 9 *AD_S.aureus_* patients, 32 *AD_control_*, and 28 *H_control_* (Table 1).

*AD*_S._a*_ureus_* patients had more severe clinical disease scores, as compared with *AD_control_* patients (mean Eczema Area Severity Index [EASI] 29.4–32 compared with an EASI of 14.8–15 in the *AD_S.aureus_* vs. *AD_control_*). Greater numbers of patients in the *AD_S.aureus_* group exhibited high levels of colonization compared with the *AD_control_* group in both the lesional (67% vs. 37%, respectively) and nonlesional (30% vs. 5%, respectively) skin. All patients in the *AD_S.aureus_* group had moderate or high levels of *S*. *aureus* on lesional skin, 60% had colonization of nonlesional skin, and 66% had nasal colonization (Table 2). In contrast, patients in the *AD_control_* group did not have clinical signs of *S*. *aureus* infection. Of these patients, 51% had *S*. *aureus* identified on lesional AD skin, falling to 30% on nonlesional AD skin, and 48% had nasal colonization. *AD_S.aureus_* patients were less likely to be on immunomodulatory systemic medication (8%–11% in *AD_S.aureus_* compared with 41%–46% in *AD_control_*) (Table 1). However, there was no significant difference between the proportion of colonized versus noncolonized AD patients on systemic immunomodulatory therapy, 21 out of 22 of whom were in the *AD_control_* group (Supplemental Figure 1A; supplemental material available online with this article; https://doi.org/10.1172/jci.insight.178789DS1). Interestingly, there was also no correlation between EASI score and the burden of *S*. *aureus* colonization in the 22 patients on immunomodulatory therapy (Supplemental Figure 1B). This suggests that systemic immunomodulatory therapy in AD can lessen the clinical impact of *S*. *aureus* colonization. Only 3% of the *H_control_* group had *S*. *aureus* skin colonization and 11% had nasal colonization (Table 2).

### Characterization of circulating T cell and S. aureus antigen–specific T cell responses associated with S. aureus–infected flares.

To determine whether differential circulating T cell phenotypes and *S*. *aureus* antigen–specific T cell responses could be identified between *AD_S.aureus_*, *AD_control_*, and *H_control_*, peripheral blood mononuclear cells (PBMCs) were collected from all 93 recruited patients. Circulating leucocyte populations were profiled to assess the proportions of specified T cell subsets by flow cytometry staining (Table 3). To assess *S*. *aureus* antigen–specific systemic responses, total PMBCs were labeled with carboxyfluorescein succinimidyl ester (CFSE) and cultured in vitro in the presence of heat-inactivated *S*. *aureus* (strain AD08). Cells stimulated with media alone or staphylococcal enterotoxin A (SEA) represented negative and positive controls, respectively. On day 8, cells were collected and stained with a panel of fluorochrome-conjugated antibodies against surface and intracellular markers (Table 4). Fluorescence-activated cell sorting (FACS), using fluorescence-minus-one controls for gating, was used to identify the specified T cell subsets. Gating strategies are described in Supplemental Figures 2–4.

**Figure 1:**
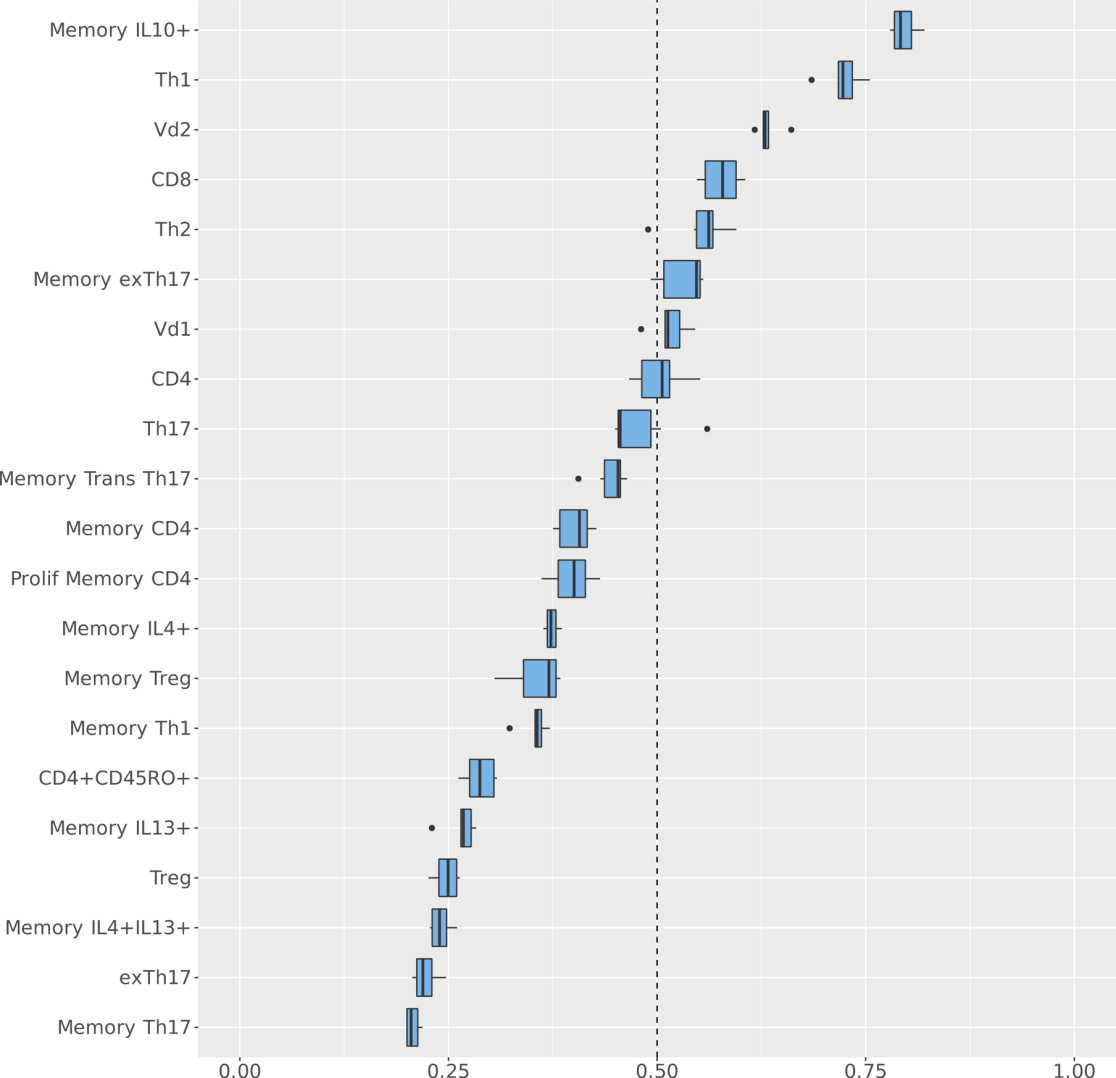
Systemic immunological variables distinguishing *AD_S.aureus_* from *AD_control_* and *H_control_*. Circulating T cell profiles and *Staphylococcus* *aureus* antigen–specific T cell memory responses from the total cohort of *n =* 93 were assessed in *AD_S.aureus_* (*n =* 12), *AD_control_* (*n =* 46), and *H_control_* (*n =* 35). Values for the circulating T cell (absence of a prefix) and *S*. *aureus* antigen–specific T cell memory responses (labeled with the prefix “Memory”) were inputted into the Bayesian multinomial model. This model was used to identify the systemic immunological variables with the highest probability of distinguishing *AD_S.aureus_* from *AD_control_* and *H_control_*. The baseline for the Bayesian multinomial regression model was the *AD_control_* cohort. The *y* axis lists each immunological feature included in the model in descending order of probability of distinguishing *AD_S.aureus_* from *AD_control_* and *H_control_*. The *x* axis indicates the probability of association with *AD_S.aureus_* as a fraction of 1, whereby 1 = 100% probability of association.

The proportions of circulating systemic T cell subsets and *S*. *aureus* antigen–specific memory T cell subsets identified in the 3 groups were inputted into a Bayesian multinomial model. This model ranks features in order of predictive value rather than binary inclusion or exclusion outputs, mitigating the limitations associated with the relatively small sample size, coupled with the large number of features (immunological parameters) assessed. The baseline for the Bayesian multinomial regression model was the *AD_control_* cohort. This model identified the T cell subsets with the highest probability of distinguishing *AD_S.aureus_* from *AD_control_* and *H_control_* and ranked them in order of highest median probability (Figure 1). The feature with the highest probability of distinguishing *AD_S.aureus_* from *AD_control_* was memory IL-10–producing T cells, with a median probability of greater than 80%, followed by circulating Th1 cells with a median probability of 75% and circulating Vδ2^+^ cells with a median probability of 63%. Other contributory distinguishing features included circulating CD8^+^, CD4^+^, Vδ1^+^, and Th2 cells, and memory ex-Th17 cells (a functionally distinct subset of Th17 cells that no longer produce IL-17 but produce IFN-γ), which had median distinguishing probability ranges between 50% and 60%.

**Figure 2:**
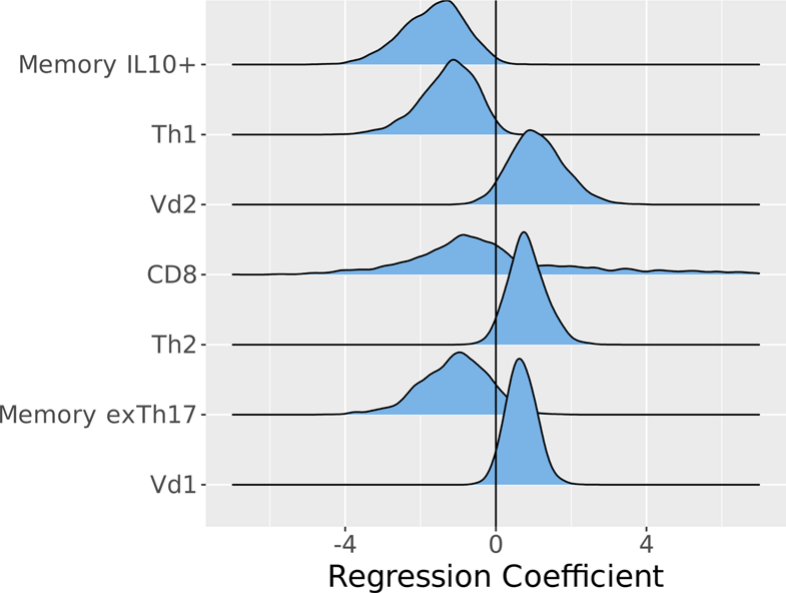
Directionality of the systemic features with the highest probability of distinguishing *AD_S.aureus_* from *AD_control_*. Ridgeplots indicate the directionality of the features from the Bayesian multinomial model, incorporating both systemic circulating T cells and systemic *Staphylococcus* *aureus* antigen–specific T cell memory responses. The central line comparator represents *AD_control_* (*n =* 46); *AD_S.aureus_* (*n =* 12) is shown in blue. The variables are ranked in descending order of median probability of distinguishing *AD_S.aureus_* from *AD_control_*. Left of the midline indicates feature suppression in *AD_S.aureus_* and right of the midline indicates increased feature expression in *AD_S.aureus_*, compared with the central comparator line, which represents *AD_control_*.

To establish the directionality of the features with the highest probability of association with *AD_S.aureus_*(>50%), ridgeplots were used to compare expression of the top 8 ranking variables in *AD_S.aureus_* as compared with *AD_control_*. Memory IL-10^+^ and circulating Th1 cells were suppressed in *AD_S.aureus_* as compared with *AD_control_*, while circulating Vδ2^+^ cells were elevated in *AD_S.aureus_* as compared with *AD_control_*. In addition, circulating CD8^+^, CD4^+^, and memory ex-Th17 cells were also suppressed in *AD_S.aureus_*, while circulating Th2 and Vδ1^+^ were elevated (Figure 2).

### Characterization of skin-homing S. aureus antigen–specific T cell responses and local skin inflammatory markers associated with S. aureus–infected flares.

The initial analysis focused on total *S*. *aureus* antigen–specific memory T cell responses. To better understand *S*. *aureus* antigen–specific memory T cell responses targeted toward the skin, cutaneous lymphocyte–associated antigen (CLA) was added to the staining panel to identify skin-homing *S*. *aureus* antigen–specific memory T cells (42). CLA is an inducible carbohydrate modification of P-selectin glycoprotein ligand-1 (PSGL1). PSGL1 is expressed constitutively on all human peripheral blood T lymphocytes (43). The CLA epitope binds specifically to E-selectin on the endothelium of postcapillary venules and allows the selective migration of T lymphocytes from the peripheral circulation to the dermis (44).

Cytokine profiles from nonlesional stratum corneum tape strips (TSs) were also obtained to identify local inflammatory responses in the skin (45). Skin cytokine profiles, in conjunction with skin-homing *S*. *aureus* antigen–specific antigen responses, were available for a subset of 69 patients (Table 3) and were analyzed alongside associated systemic circulating T cell profiles for these patients, using the Bayesian multinomial model. As before, the results identified both systemic and local cutaneous T cell–mediated immune responses, with the highest probability of distinguishing *AD_S.aureus_* from *AD_control_* and *H_control_*. The addition of skin-homing systemic responses and markers of the cutaneous inflammatory response increased the number of features, with a greater than 50% probability of distinguishing *AD_S.aureus_* from *AD_control_* and *H_control_* from 8 to 30. This indicates that these additional features provide greater insight into the site-specific immune profiles associated with *AD_S.aureus_*.

**Figure 3:**
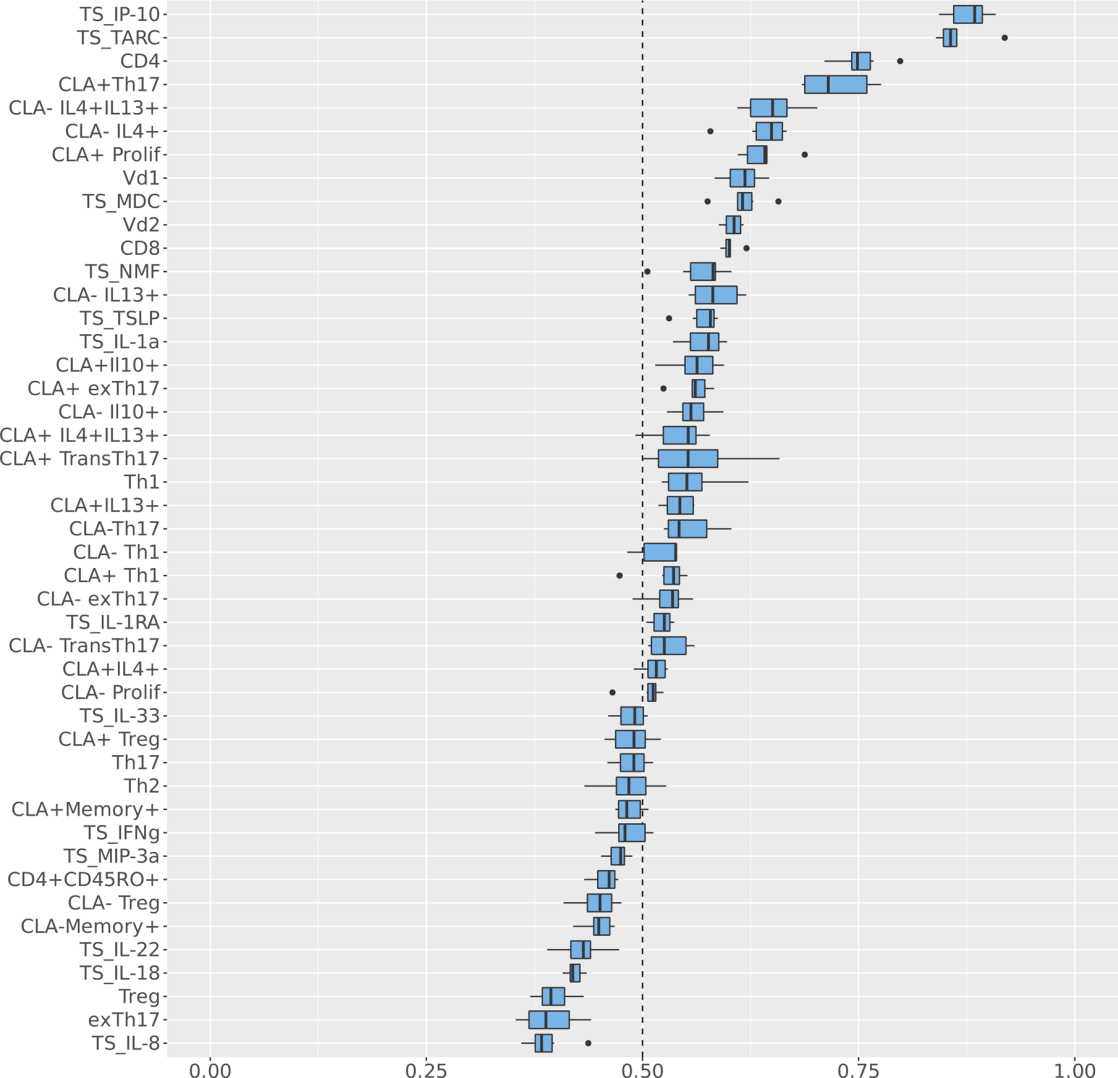
Immunological variables distinguishing *AD_S.aureus_* from *AD_control_* and *H_control_* when incorporating systemic circulating T cells, CLA^+^ and CLA^–^ *Staphylococcus* *aureus* antigen–specific T cell memory responses, and stratum corneum tape strip cytokines. Circulating T cell profiles, *S*. *aureus* antigen–specific T cell memory responses (both CLA^+^ and CLA^–^), and stratum corneum tape strip cytokines from the cohort of 69 patients for whom a complete data set was available were assessed in *AD_S.aureus_* (*n =* 9), *AD_control_* (*n =* 32), and *H_control_* (*n =* 28). Values for the circulating T cell (absence of a prefix) and *S*. *aureus* antigen–specific T cell memory responses (labeled with the prefix “Memory”) were inputted into the Bayesian multinomial model. This model was used to identify the systemic immunological variables with the highest probability of distinguishing *AD_S.aureus_* from *AD_control_* and *H_control_*. The baseline for the Bayesian multinomial regression model was the *AD_control_* cohort. The *y* axis lists each immunological feature included in the model in descending order of probability of distinguishing *AD_S.aureus_* from *AD_control_* and *H_control_*. The *x* axis indicates the probability of association with *AD_S.aureus_* as a fraction of 1, whereby 1 = 100% probability of association. CLA, cutaneous lymphocyte-associated antigen; TS, tape strip.

The features with the highest probability of distinguishing *AD_S.aureus_* from *AD_control_* and *H_control_* were TS IP10 and TARC, with a probability approaching 90%, followed by circulating CD4^+^ and CLA^+^ Th17, with a probability between 70% and 80%. Further features with a probability above 60%, corresponding with the shoulder of the curve (Figure 3), were circulating Vδ1^+^, Vδ2^+^, and CD8^+^ cells, memory CLA^–^IL-4^+^IL-13^+^ T cells, memory CLA^–^IL-4^+^ T cells, TS macrophage-derived chemokine (MDC, also known as CCL22), and memory CLA^+^ total proliferating T cells.

**Figure 4:**
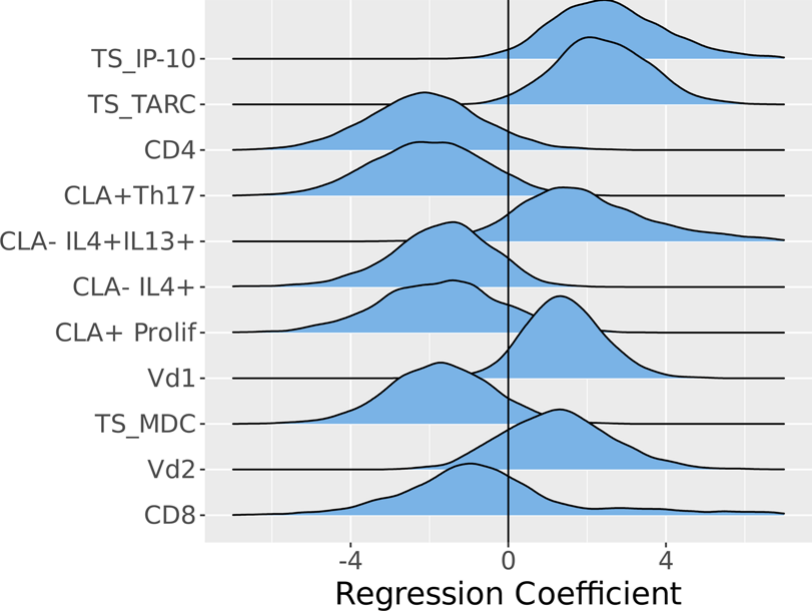
Directionality of the highest-ranking variables (incorporating systemic circulating T cells, *Staphylococcus* *aureus* antigen–specific T cell memory responses with skin-homing marker CLA, and stratum corneum tape strip cytokines) with the highest probability of distinguishing *AD_S.aureus_* from *AD_control_*. Ridgeplots show the directionality of the highest-ranking variables from the Bayesian multinomial model, incorporating systemic circulating T cells, *S*. *aureus* antigen–specific T cell memory responses with the addition of the skin homing marker CLA, and local responses obtained from tape strip cytokines. The comparator is *AD_control_* (*n =* 46). Left of the midline indicates feature suppression in *AD_S.aureus_* (*n =* 12); right of the midline indicates increased feature expression in *AD_S.aureus_* as compared with the central line comparator, which represents *AD_control_*. Total PBMCs were isolated from whole blood by density gradient centrifugation. Circulating T cell profiles were identified from FACS analysis of PBMCs. *S*. *aureus* antigen–specific T cell memory responses were identified following the incubation of PBMCs with heat-killed *S*. *aureus* strain AD08. FACS analysis was used to identify *S*. *aureus* antigen–specific T cell proliferation and cytokine production, with the inclusion of the skin-homing marker, CLA. Cytokines were extracted from tape strips, measured on multiplex panels, and normalized for stratum corneum protein content. Values for the circulating T cell and *S*. *aureus* antigen–specific T cell memory responses were inputted into a combined multinomial Bayesian model. Absence of a prefix indicates subtype is a circulating T cell. CLA, cutaneous lymphocyte associated antigen; Memory, *S*. *aureus* antigen–specific memory responses; TS, tape strip.

To establish the directionality of the top-ranking features with the highest probability of association with *AD_S.aureus_*, ridgeplots were utilized. Both TS IP10 and TARC were elevated in *AD_S.aureus_* as compared with AD_control_, along with memory CLA^–^IL-4^+^IL-13^+^ T cells and the circulating γδ T cell subsets, Vδ1^+^ and Vδ2^+^. In contrast, circulating CD4^+^, CD8^+^, proliferating CLA^+^ memory T cells, memory CLA^+^ Th17, memory CLA^–^IL-4^+^, and TS MDC were lower in *AD_S.aureus_* compared with *AD_control_* (Figure 4). The fact that 2 TS variables had the highest probability of distinguishing *AD_S.aureus_* from *AD_control_* and *H_control_* is consistent with a skin-dominant inflammatory signal, as expected in primary skin infection. The highest-ranking variable, TS IP10 (CXCL10), is a member of the CXC chemokine subfamily and is an important chemokine for attracting primarily Th1 lymphocytes and neutrophils (46). Upregulation of CXCL10, a ligand of the CXCR3 receptor, has also been shown to promote chronic itch in AD (47). The next highest-ranking variable was TARC (CCL17). TARC is a CC chemokine and a ligand for CCR4 (C-C chemokine receptor type 4) found on Th2 and Th17 cells (48), but is primarily a chemoattractant for skin-homing Th2 lymphocytes in AD (49). MDC (CCL22) expression also had a higher probability of association with *AD_S.aureus_*. MDC is a CC chemokine that also acts as a chemoattractant for CCR4-expressing cells. Higher levels of IP10 and TARC expression in *AD_S.aureus_* compared with *AD_control_* suggest that Th1, Th2, and potentially Th17 cells are directed toward the skin compartment in the setting of an acute *S*. *aureus* infection in AD (48).

Despite the addition of TS and skin-homing variables to our model, many T cell markers remained consistently present among the highest-ranking variables. These included circulating CD4^+^, CD8^+^, Vδ1^+^, and Vδ2^+^. There was again global suppression of CD4^+^ and CD8^+^ expression in conjunction with the additional finding of suppressed memory CLA^+^ proliferating T cells. These findings support a potential role for *S*. *aureus*–mediated immunosuppression, inhibiting protective host T cell responses in *AD_S.aureus_*. Similar to the systemic-only analysis, circulating Vδ1^+^ and Vδ2^+^ expansion again had a high probability of association with the *AD_S.aureus_* cohort, which may represent a compensatory immune response mechanism in the setting of αβ T cell suppression. Longitudinal analysis extending to the postconvalescent phase is required to confirm whether these findings represent immunomodulation secondary to *S*. *aureus* infection or underlying host aberrant T cell profiles.

The addition of CLA to the analysis revealed a high probability of memory CLA^+^ Th17 cell suppression and memory CLA^–^IL-4^+^IL-13^+^ expansion (a marker of Th2 cell activity) in *AD_S.aureus_*. These findings provide greater insights into the systemic T cell responses specifically directed toward the skin compartment. The reduced memory CLA^+^ Th17 cell response identified in *AD_S.aureus_* may also represent skin homing of this lymphocyte subset from the systemic circulation in response to *S*. *aureus* skin infection.

The expansion of both circulating Th2 cells in the systemic-only analysis and in the *S*. *aureus* antigen–specific IL-4^+^IL-13^+^ (Th2) memory response in the combined analysis reflects the Th2 signature characteristic of AD (50) amplified *by S*. *aureus* (51, 52). The memory CLA^–^IL-4^+^IL-13^+^ *S*. *aureus* antigen–specific Th2 response had a higher probability of association with *AD_S.aureus_*. This may be due to skin homing by the CLA^+^IL-4^+^IL-13^+^ population. Interestingly, only dual IL-4– and IL-13–producing T lymphocytes were consistently elevated in *AD_S.aureus_* as compared with *AD_control_*.

### Adjustment for systemic immunomodulatory therapy.

**Figure 5:**
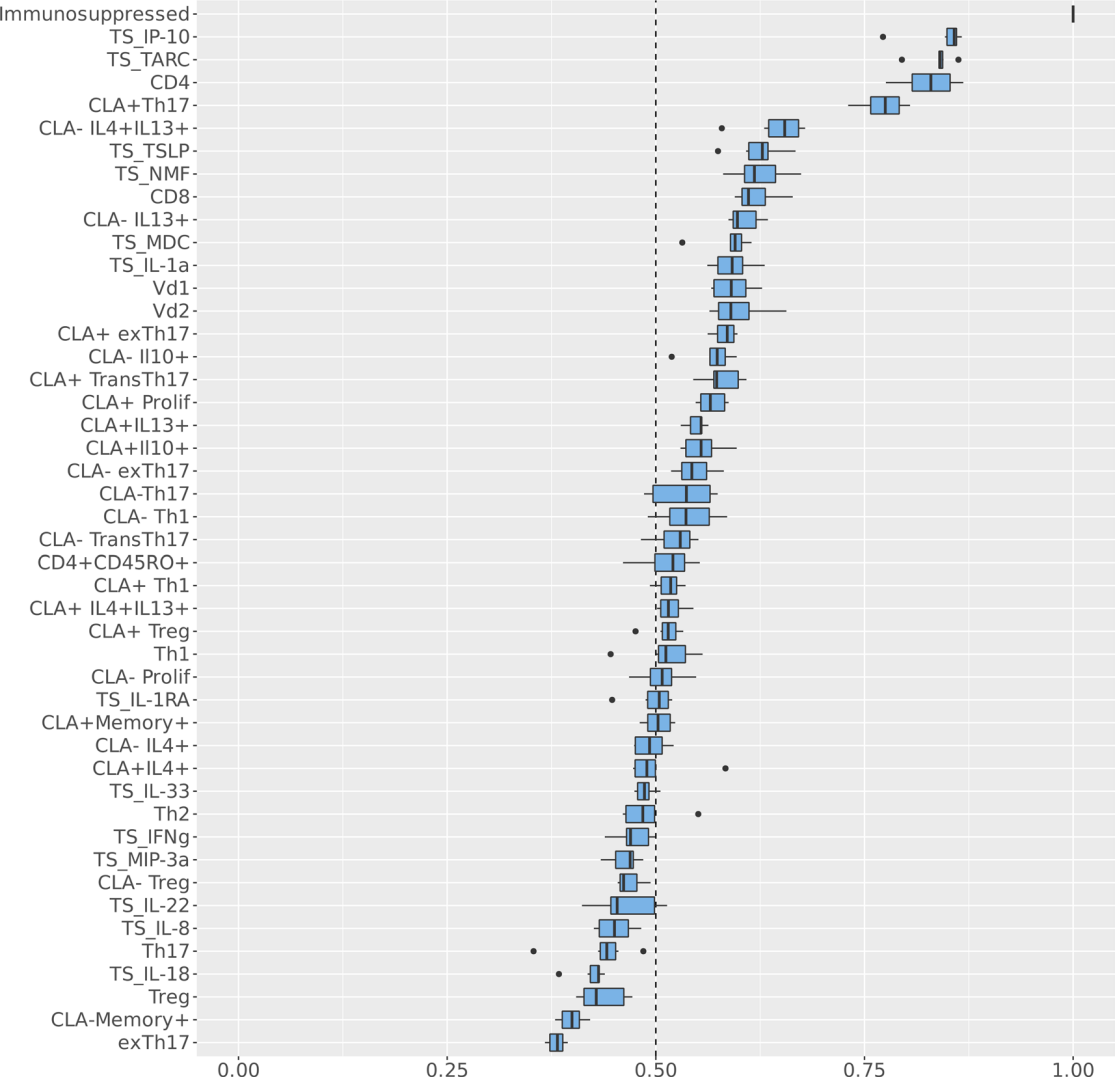
Immunological variables distinguishing *AD_S.aureus_* from *AD_control_* and *H_control_* when incorporating systemic circulating T cells, CLA^+^ and CLA^–^ *Staphylococcus* *aureus* antigen–specific T cell memory responses, and stratum corneum tape strip cytokines and an adjustment variable for systemic immunomodulatory therapy. Circulating T cell profiles, *S*. *aureus* antigen–specific T cell memory responses (both CLA^+^ and CLA^–^), and stratum corneum tape strip cytokines from the cohort of 69 patients for whom a complete data set was available were assessed in *AD_S.aureus_* (*n =* 9), *AD_control_* (*n =* 32), and *H_control_* (*n =* 28). The adjustment variable for systemic immunomodulatory therapy is listed above the immunological features. It is a mandatory feature for this selected cohort ensuring that all iterations in the adjusted model account for the effects of systemic immunomodulation. Values for the circulating T cell (absence of a prefix) and *S*. *aureus* antigen–specific T cell memory responses (labeled with the prefix “Memory”) were inputted into the Bayesian multinomial model. This model was used to identify the systemic immunological variables with the highest probability of distinguishing *AD_S.aureus_* from *AD_control_* and *H_control_*. The baseline for the Bayesian multinomial regression model was the *AD_control_* cohort. The *y* axis lists each immunological feature included in the model in descending order of probability of distinguishing *AD_S.aureus_* from *AD_control_* and *H_control_*. The *x* axis indicates the probability of association with *AD_S.aureus_* as a fraction of 1, whereby 1 = 100% probability of association. CLA, cutaneous lymphocyte associated antigen; TS, tape strip.

Systemic immunomodulatory therapy is used in clinical practice to treat AD refractory to standard topical regimens. These medications act on multiple inflammatory pathways, including T lymphocyte–mediated responses, to limit disease severity. Within the combined skin and systemic immune profiling analysis subcohort, 1 patient in the *AD_S.aureus_* group and 21 patients in the *AD_control_* group were on systemic immunomodulatory therapy for AD (Table 3), predominantly methotrexate. An adjustment variable was therefore added to the model to account for the effects of immunomodulatory therapy, as described in the Methods. Importantly, the top 5 variables with the highest probability of distinguishing *AD_S.aureus_* from *AD_control_* and *H_control_* remained unchanged between the analyses adjusted for systemic therapy and nonadjusted analyses (Figure 5). These variables are TS IP10, TS TARC, circulating CD4^+^, memory CLA^+^ Th17, and CLA^–^IL-4^+^IL-13^+^ T cells. This indicates that the strongest immune signals identified arise independently of the effects of systemic immunosuppressants.

Two additional markers with an associated probability with *AD_S.aureus_* of greater than 60%, which differs from the unadjusted analysis, are TS thymic stromal lymphoprotein (TSLP) and TS natural moisturizing factor (NMF). Like TARC, TSLP is a cytokine produced by epithelial cells, keratinocytes, and dendritic cells that drives Th2 responses (53). NMF is a TS-derived biomarker. It is a skin humectant composed of filaggrin degradation products and is often determined as a surrogate marker of *FLG* gene mutation status (54). However, in AD the NMF levels are strongly influenced by Th2 inflammation, which downregulates filaggrin expression (55). NMF had a median probability of distinguishing *AD_S.aureus_* from *AD_control_* of greater than 60% when factoring in the effects of systemic immunomodulatory therapy. Features from the unadjusted analysis that have a probability below the selected threshold of greater than 60% in the adjusted analysis include Vδ1^+^ and Vδ2^+^, CLA^+^ proliferating memory T cells, memory CLA^–^IL4^+^ T cells, and TS MDC. Overall, however, this adjusted analysis suggests that immunosuppression does not affect the main distinguishing variables associated with *AD_S.aureus_*.

## Discussion

**Figure 6:**
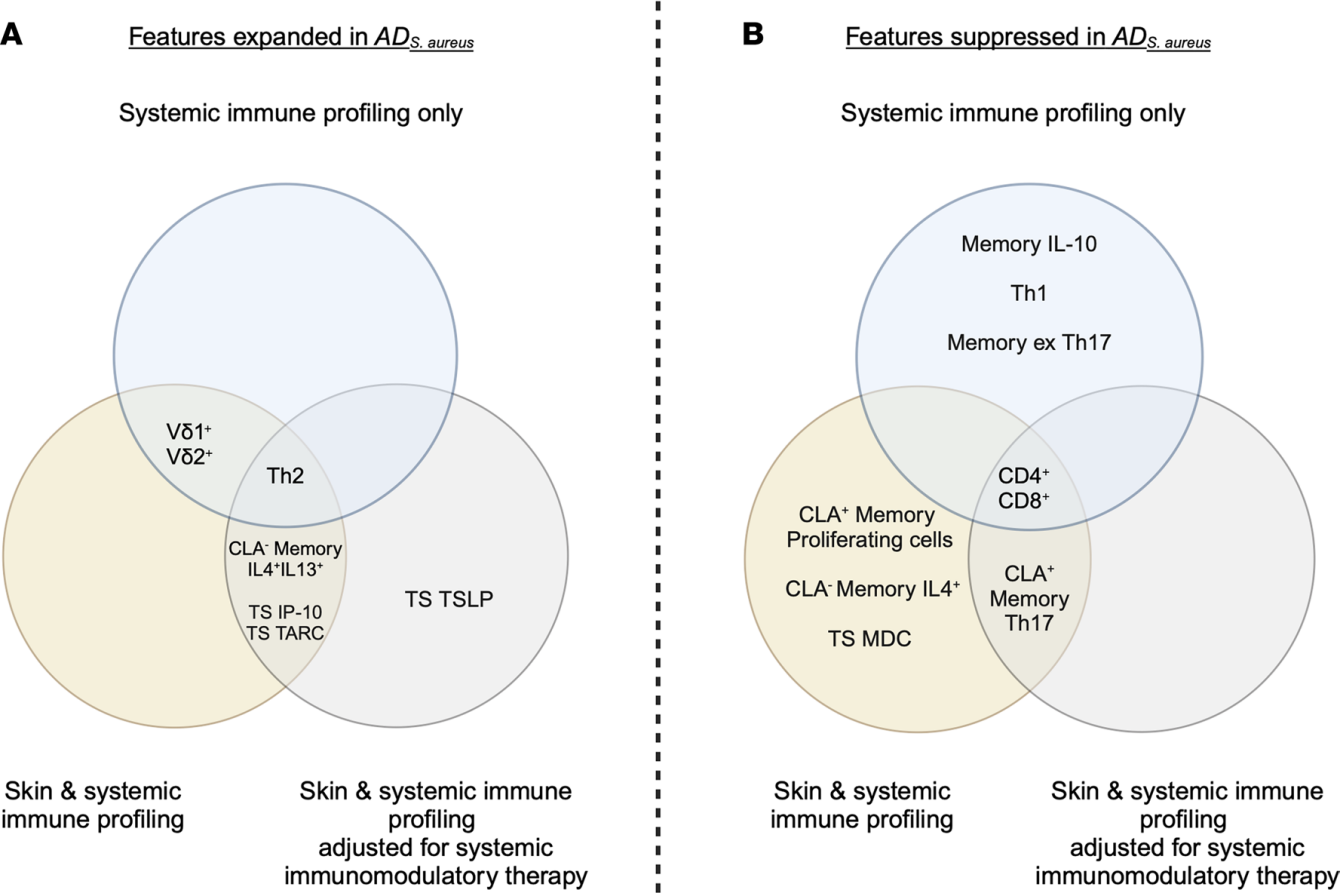
Features expanded and suppressed in *AD_S.aureus_*. Venn diagrams outline the features expanded (**A**) and suppressed (**B**) in *AD_S.aureus_* in the Bayesian multinomial models. The blue circle represents the analysis of systemic immune profiles only, the yellow circle represents the combined skin and systemic immune profiling, and the gray circle represents the combined skin and systemic immune profiling adjusted for systemic immunosuppressive treatments of AD. For systemic immune profiling there were a total of *n =* 93 patients, with *n =* 12 in the *AD_S.aureus_* group. For combined skin and systemic immune profiling and combined skin and systemic immune profiling adjusted for systemic immunosuppressive treatments, there were a total of *n =* 69 patients, with *n =* 9 in the *AD_S.aureus_* group. Absence of a prefix indicates subtype is a circulating T cell. CLA, cutaneous lymphocyte-associated antigen; Memory, *S*. *aureus* antigen–specific memory responses; TS, tape strip.

This study provides insights into the systemic and cutaneous immune signatures associated with *S*. *aureus*–infected pediatric AD (*AD_S.aureus_*) (Figure 6) using an innovative Bayesian multinomial model combined with a feature selection method. An analysis of the systemic-only response to *AD_S.aureus_* identified lower numbers of of circulating Th1, CD4^+^, CD8^+^, and *S*. *aureus* antigen–specific memory IL-10^+^ and ex-Th17 cells, alongside increased circulating Th2 and the γδ T cell subsets Vδ1^+^ and Vδ2^+^. When the analysis was expanded to include stratum corneum TS cytokines and CLA-specific skin-homing memory T cell responses, this identified elevated expression of TS cytokines IP10 and TARC and reduced expression of MDC, which influence T cell migration from the systemic circulation to the skin in AD. The addition of CLA identified memory CLA^+^ Th17 suppression and memory CLA^–^IL-4^+^IL-13^+^ (Th2) expansion as additional mediators in the skin-homing response in patients with *S*. *aureus* skin infection.

Stratum corneum nonlesional TS biomarkers, namely IP10 and TARC, had the highest probability of distinguishing *AD_S.aureus_* from *AD_control_* and *H__control__,* approaching 90%. This is consistent with a dominant cutaneous inflammatory signal, consisting predominantly of Th1, Th2, and potentially also Th17 cells, in the setting of an acute *S*. *aureus* skin infection in this AD cohort. IP10/CXCL10 attracts Th1 lymphocytes and neutrophils (46), acting as a ligand for CXCR3 on Th1 cells (56). CXCL10/CXCR3 signaling is known to promote pruritus via activation of sensory neurons (47). This finding supports the clinical manifestations of worsening itch associated with AD flares. Elevated IP10 from lesional and nonlesional stratum corneum TSs in AD has been described in adult AD; however, the presence of *S*. *aureus* skin colonization or infection was not specified (57). Our data suggest upregulation of IP10 is associated with *S*. *aureus* skin infection in AD; however, longitudinal assessment after infection is required for confirmation. Given the very high probability of the association of IP10 with *AD_S.aureus_*, however, our findings suggest it is a discriminatory feature between *AD_S.aureus_* and *AD_control_* in this cohort. TARC, also referred to as CCL17, is a ligand for CCR4, found on Th2 and Th17 lymphocytes. As CCR4 is coexpressed by the majority of CLA^^+^^ lymphocytes, TARC is a chemoattractant for Th2/Th17 skin-homing lymphocytes, but is predominantly associated with activation of Th2 signaling in AD (49). Serum TARC has also been identified as a biomarker of clinical severity in AD (58). Both lesional (57) and nonlesional stratum corneum TS TARC values have been shown to correlate with AD severity scores (59). We show a specific association with elevated TARC expression and *S*. *aureus* skin infection in AD. MDC (CCL22) is also a ligand for CCR4; however, MDC expression was suppressed in *AD_S.aureus_* compared with *AD_control_*, but overall was more highly expressed in our AD cohort compared with *H_control_* (60). TSLP expression, which preferentially drives Th2 responses, was also increased in *AD_S.aureus_* (53). Overall, this pattern is consistent with a dominant Th1/Th2 cutaneous immune profile in *AD_S.aureus_*, but also suggests Th17 activation is present as expected (61), albeit to a lesser degree.

The remaining results reveal an overall pattern of host systemic immunosuppression associated with *AD_S.aureus_* (Figure 6). This is characterized by global suppression of circulating CD4^^+^^ and CD8^^+^^ αβ T cell responses across each iteration of the Bayesian analysis. Circulating Th1 lymphocytes and ex-Th17 cells, both involved in protective systemic responses to *S*. *aureus* infection (62, 63), were suppressed in *AD_S.aureus_*. The observed suppression of memory IL-10^^+^^ T cell responses in the *AD_S.aureus_* cohort may also reflect inhibition of polyclonal T cells capable of producing IFN-γ and IL-10 (64). In contrast, circulating Th2 and memory Th2 responses, associated with atopy and a tolerogenic environment for *S*. *aureus*, were enhanced. Although Th2 responses were elevated in AD, only dual IL-4– and IL-13–producing T lymphocytes were consistently elevated in *AD_S.aureus_* as compared with *AD_control_*.

Protective systemic CD4^^+^^ *S*. *aureus* antigen–specific T cell proliferating memory responses were suppressed in *AD_S.aureus_*, most notably the CLA^^+^^ Th17 memory response; however, this may also represent skin homing of this population from the systemic circulation. IL-17, produced by Th17 cells, promotes neutrophil recruitment and upregulates keratinocyte expression of antimicrobial peptides (65) and is critical for generating immune responses at epithelial sites (66). Suppressed antigen-specific T cell responses in primary *S*. *aureus* skin infection have previously been observed in a non-AD mouse model (67). In pediatric AD, skin colonization with *S*. *aureus* has been associated with reduced IFN-γ production from CD4^^+^^ PBMCs in response to nonspecific stimulation (68). *S*. *aureus* antigen–specific T cell responses to *S*. *aureus* colonization and infection, however, have not been previously identified.

Although this study suggests a pattern of host immunosuppression associated with AD, due to the study’s cross-sectional design, it is not possible to confirm whether this arises as a cause or a consequence of *S*. *aureus* skin infection. We hypothesize that this may represent *S*. *aureus*–mediated immunosuppression in the *AD_S.aureus_* cohort, with greater inhibition of protective host T cell responses, owing to existing knowledge of *S*. *aureus*–mediated manipulation of host immunity (69). Staphylococcal superantigens are known to have the potential to inhibit antigen-specific proliferation of T cells, leading to an anergic response (70). In addition, an *S*. *aureus–*derived toxin, δ toxin, can directly lyse T cells (71) and trigger mast cell degranulation, a factor in the exacerbation of *S*. *aureus*–infected AD lesions (72). The observed αβ T lymphocyte suppression distinguishes *AD_S.aureus_* from *AD_control_*, despite the fact that 51% of *AD_control_* patients were colonized by *S*. *aureus*. These findings point toward an important role for altered adaptive immunity in the shift from *S*. *aureus* colonization to clinical infection in AD. Only one patient within the *AD_S.aureus_* cohort was on systemic immunomodulatory therapy, diminishing the potential contributory role of iatrogenic immunosuppression. This likely suggests either intrinsic host immunocompromise specific to the *AD_S.aureus_* cohort or a pathogen-mediated immunosuppressive effect in the context of active infection.

In contrast to the overall picture of host systemic immunosuppression, we identify a niche role for Vδ1^^+^^ and Vδ2^^+^^ γδ T cell expansion in *S*. *aureus*–infected AD. Small observational studies on the role of Vδ1^^+^^ and Vδ2^^+^^ γδ T cells in AD show conflicting results, with both suppression (73, 74) and expansion (75) of γδ T cells described. It is known that human Vδ2^^+^^ cells are activated by *S*. *aureus*–infected dendritic cells to produce IFN-γ, which plays a key role in anti-staphylococcal immunity (62). We hypothesize that Vδ2^^+^^ expansion observed in *AD_S.aureus_* reflects a host compensatory response in the setting of αβ T cell suppression. A similar phenomenon has previously been described in IRAK4/MyD88-deficient individuals, whereby Vδ2^^+^^ γδ T expansion acts to restore an otherwise impaired neutrophil response (76).

A substantially higher proportion of patients in the *AD_control_* group were treated with systemic immunomodulatory medication, as compared with the *AD_S.aureus_* group (41% vs. 11% in the systemic and skin profiling analysis). An adjustment to the model, accounting for the effects of systemic immunomodulatory therapies used (predominantly methotrexate), demonstrated that the top 5 variables with the highest probability of distinguishing *AD_S.aureus_* were unchanged. This suggests that immunosuppression did not affect the main distinguishing variables associated with *AD_S.aureus_*, of which skin homing Th17 and Th2 cells are key. The lower prevalence of *S*. *aureus* infection and burden of *S*. *aureus* colonization among *AD_control_* as compared with *AD_S.aureus_* suggests that immunomodulation is a reasonable treatment approach to limit *S*. *aureus*–mediated flares of AD until more targeted therapeutics are developed.

Tissue- or site-specific immunity is an important element of *S*. *aureus* vaccine design, as host responses vary depending on the primary infection site. Previous vaccine studies in humans have focused primarily on systemic responses (36, 37); however, the local cutaneous response is of particular relevance in AD. Our approach, which examined systemic T cell responses prior to the addition of cutaneous and skin-homing signaling, highlights the utility of a sequential method in identifying systemic signals that could otherwise be masked by strong cutaneous cytokine signaling. The addition of the skin-homing marker CLA provides important insights into the role for T cell responses to *S*. *aureus* skin infection, not otherwise apparent in an analysis of systemic-only responses. Both Bayesian multinomial models and the feature selection algorithm we use are well-established statistical methods, although these are generally not utilized in a combined approach. An advantage of the method used in this study is the ability of the latent variable approach to rank variables in order of their probability of association with the outcome measure, namely *S*. *aureus* infection in AD. A similar but nonidentical model has been recently reported (77); however, to the best of our knowledge we describe the first known use in this format. Our approach allows for the identification of subtle immunological signals within complex biological systems. This may have broader applicability in similar data sets, analyzing multiple immunological and clinical features.

An important consideration in anti–*S*. *aureus* vaccine design is the timing of administration in infancy, childhood, or later life. Earlier administration could potentially mitigate the more severe manifestations of AD. Vaccine administration prior to *S*. *aureus* exposure, however, would be extremely challenging. Therefore, a potential vaccine would likely need to counteract *S*. *aureus*–mediated immunomodulatory effects (78), assuming prior colonization and/or infection.

Limitations of the study include the relatively small sample size, which allows us to illustrate data trends but limits the generalizability of our statistical model’s predictive performance. The model identifies multiple immunological features associated with *AD_S.aureus_*; however, these findings need to be replicated in larger studies. Longitudinal analysis of the *AD_S.aureus_* cohort after convalescence is ultimately required to support or refute the hypothesis that the immunological signatures observed arise as a consequence of infection. Furthermore, the study cohort consists solely of pediatric patients whose immunological profiles differ from those associated with adult AD and cannot be directly extrapolated to this population (79). Previous anti–*S*. *aureus* vaccines have been targeted toward high-risk adult populations. Given the high prevalence of AD among the pediatric population and the observed immune dysregulation in association with *S*. *aureus* in this study, pediatric patients merit greater consideration in the design of future anti–*S*. *aureus* vaccines.

Future directions for this work include expansion to a larger patient cohort over a prolonged time course, with greater ethnic diversity, age range, and stratifying patient cohorts according to age profiles, genotypic, and endotypic features, including filaggrin and IgE status. This is essential to identify heterogeneous responses, which may affect vaccine therapeutic responses. It will also be important to investigate *S*. *aureus*–specific CD4^^+^^ skin tissue–resident memory T (Trm) cells. These cells have recently been described in healthy abdominal skin explants (80) and are likely to play a substantive role in mediating local cutaneous responses to *S*. *aureus* in AD. Due to the limited feasibility of obtaining these samples in a pediatric population, future studies may require the use of human 3D skin models (81).

In summary, this study provides what we believe are novel data on the cutaneous and systemic immunological profiles associated with *S*. *aureus* skin infection in AD. We identify an overall pattern of systemic host immunosuppression associated with *AD_S.aureus_* manifesting as suppression of αβ T cell and protective *S*. *aureus* antigen–specific T cell proliferating memory responses, in particular CLA^^+^^ Th17. Potentially, this cohort may represent a subset of AD patients susceptible to *S*. *aureus* infection due to intrinsic immune dysregulation. We hypothesize, however, that our observations represent pathogen-mediated effects unmasked in the shift from *S*. *aureus* colonization to infection. Further work is required to identify the exact pathomechanism and to determine the relative contribution of host versus bacteria in the observed suppression of conventional protective adaptive immune responses. We also identify a role for γδ T cells as a potential anti–*S*. *aureus* vaccine target promoting protective host immune responses. A comprehensive understanding of the host immune response to *S*. *aureus* has the potential to revolutionize treatment approaches in the management of AD. This is a key area for continued research with substantial potential translational impact.

## Methods

### Sex as a biological variable.

Our study included male and female human patients, and groupings overall were closely matched for sex. We do not report sex-stratified analyses, as sex has to date not been identified as a significant factor affecting AD severity in the pediatric population (82).

### Patient recruitment.

Ninety-three patients aged 0–16 years were recruited from a single tertiary pediatric center from January to March 2020 and from July 2020 to March 2021. There were 58 patients with AD according to the Hanifin and Rajka diagnostic criteria for AD (83) and 35 healthy control participants. *S*. *aureus* skin infection was determined by clinical features on examination (3) identified by the recruiting physician and supervising pediatric consultant dermatologist, with confirmatory skin swabs positively identifying *S*. *aureus*. Relevant clinical features of *S*. *aureus* infection included honey-colored crusts, skin weeping, and pustules in conjunction with signs of an AD flare such as increased erythema, edema, papulation, and excoriation. Healthy controls were age- and sex-matched patients who did not have a history of atopy and did not have clinical signs or symptoms of an active *S*. *aureus* skin infection. Exclusion criteria included any other active infection or febrile illness within the past 14 days, coexistent primary immunodeficiency, or administration of oral glucocorticoids within the past 30 days. There were no exclusions based on age, sex, ethnicity, disease severity, or other nonglucocorticoid systemic immunomodulatory medication prescribed for the management of AD. Disease severity was assessed using the EASI (84) for objective real-time assessment, and the Nottingham Eczema Severity Score (NESS) (85) was used to determine disease activity in the preceding 12 months.

### Assessment of S. aureus colonization.

Bacterial swabs were obtained from the nose and nonlesional skin in all AD patients and healthy controls and from lesional AD skin in all AD patients. Staphylococci were isolated from swabs on CHROMagar Staphylococcus and Mannitol Salt Agar (MSA; Fannin). A portion of the 16S rRNA gene was amplified from individual colonies, using primers 16S3 up and 16S3 down and DNA sequencing of the amplimer was undertaken as previously described (86). Using this sequence, strains were identified at the species level using a standard nucleotide BLAST search. The bacterial burden on swabs was quantified as follows. The swab was used to lawn an entire CHROMagar MSA plate. After incubation at 37°C for 18–24 hours, the number of *S*. *aureus* colonies was counted and the load assessed as follows: low, 5 or fewer colonies; medium, 6–40 colonies; high, 41–299 colonies; very high, 300 or more colonies or complete covering of the plate.

### Preparation of heat-inactivated S. aureus bacteria.

*S*. *aureus* strain AD08, a strain representative of clonal complex 1, the most prevalent clonal complex previously identified within our patient cohort (87), was cultured overnight in Tryptic Soy Broth (TSB) (Fannin). Bacteria were suspended in sterile PBS (Sigma-Aldrich), diluted to 1 × 10^8^ CFU/mL based on optical density measurements, and heat inactivated in a dry block heater at 90°C for 45 minutes. The suspensions were centrifuged and washed in sterile PBS to remove secreted proteins and resuspended for use. The Pierce Micro BCA Protein Assay Kit (Thermo Fisher Scientific) was used to determine total protein concentration in each stock solution to ensure a standard quantity of protein was added to each assay.

### Isolation of PBMCs.

Peripheral blood was collected using an aseptic technique compliant with safe limits for child health research (88). All samples were processed within 24 hours of venipuncture, at room temperature, under sterile conditions (Supplemental Data Set 5 – MIATA/MIANKA checklist). PBMCs were isolated by density gradient centrifugation using Ficoll (Lymphoprep, Serumwerk Bernburg AG).

### Flow cytometric analysis of baseline circulating T cells.

Total PBMCs were stained with extracellular surface fluorochrome-conjugated antibodies against CD3 (PerCP-Cy5.5; clone OKT3; eBioscience), CD4 (PE-Cy7; clone RPA-T4; eBioscience), CD8 (BV510; clone SK1; BioLegend), CD45RO (PE; clone UCHL1; eBioscience), Vδ1 (APC; clone REA173; Miltenyi Biotec), Vδ2 (BV711; clone B6; BioLegend), CD25 (BB515; clone 2A3; BD Biosciences), CD127 (APC-eF780; clone eBioRDR5; eBioscience), CD161 (PE-Cy5; clone DX12; BD Biosciences), CXCR3 (PE-CF594; clone 1C6/CXCR3; BD Biosciences), CCR4 (BV421; clone 1G1; BD Biosciences), and CCR6 (BV786; clone 11A9; BD Biosciences) to identify individual leukocyte populations (Table 1). Flow cytometric data were acquired with a BD LSR Fortessa using Diva software (BD Biosciences) and analyzed using FlowJo software (Tree Star, Inc). Fluorescence-minus-one controls were used for gating. Gating strategies are described in Supplemental Figures 2–4.

### Assessment of circulating S. aureus antigen–specific T cells.

PBMCs were labeled with CFSE (Invitrogen/Thermo Fisher Scientific) and were cultured in complete RPMI 1640 (Sigma-Aldrich) supplemented with 10% heat-inactivated FBS (Sigma-Aldrich), 2 mM L-glutamine (Sigma-Aldrich), 100 U/mL penicillin, and 1 mg/mL streptomycin (Sigma-Aldrich) either alone (negative control) or in the presence of SEA (Sigma-Aldrich) at a concentration of 1 μg/mL (positive control), or heat-inactivated *S*. *aureus* strain AD08, 25 μg/mL. Cells (2 × 10^5^ per well) were set up for each condition (10 wells per condition). Cells were incubated at 37°C and 5% CO_2_. On day 8, phorbol 12-myristate 13-acetate (PMA) (50 ng/mL: Sigma-Aldrich), ionomycin (500 ng/mL; Sigma-Aldrich), and Brefeldin A (5 μg/mL; VWR) were added to the cultures for the final 4 hours. Cells were then harvested by centrifugation before extracellular and intracellular staining. Cells were resuspended in 1:1000 dilution of Fixable Viability Stain (Life Technologies/Thermo Fisher Scientific) for live/dead cell determination. Cells were subsequently resuspended in 10% BSA (Sigma- Aldrich) and stained with fluorochrome-conjugated antibodies against CD4 (PE-Cy7; clone RPA-T4; eBioscience), CD45RO (PE; clone UCHL1; eBioscience), CD161 (PE-Cy5; clone DX12; BD Biosciences), CD25 (BV605; clone BC96; BioLegend), CD127 (BV711; clone A019D5; BioLegend), and CLA (BV-650; clone M-A251; BD Biosciences). Cells were then fixed and permeabilized using the Fix and Perm Kit (Life Technologies) before intracellular staining with fluorochrome-conjugated antibodies against IFN-γ (APC-eF780; clone 4S.B3; eBioscience), TNF-α (AF700; clone Mab11, eBioscience), IL-17A (BV 421; clone B-D48; BD Biosciences), IL-4 (BV 786; clone MP4-25D2; BD Biosciences), IL-10 (PE-CF594; clone JES3-19F1; BD Biosciences), and IL-13 (APC; clone JES10-5A2, BD Biosciences) to identify expansion of antigen-specific T cell subsets (Table 4). Cells were analyzed by flow cytometry as described above. Gating strategies are described in Supplemental Figures 2–4. Values were corrected for background proliferation by subtracting response to negative unstimulated control cells.

### Analysis of skin cytokine levels and NMF.

Stratum corneum was sampled using circular adhesive tapes (22-mm diameter D-Squame Discs; CuDerm Corporation). The tapes were placed on nonlesional forearm skin of patients and healthy controls as previously described (90). Adhesive tapes were pressed for 10 seconds with a pressure of 225 g/cm, using a D-Squame Pressure Instrument D500 (CuDerm Corporation). Sequentially, 20 consecutive TSs were collected from the same skin site, placed individually in 2-mL cryovials, and immediately stored at –80°C. Cytokine expression in the stratum corneum was quantified as previously described (89). Briefly, PBS (Merck) and 0.005% Tween 20 (Sigma-Aldrich) were added to each cryovial and left on ice for 30 minutes. Extraction was performed for 15 minutes with an ultrasound sonifier (Branson 5800). The extract was centrifuged and supernatant aliquots of 60 μL were frozen at –80°C until further analysis. The amount of cytokine in the stratum corneum was normalized for the protein content, which was determined using the Pierce Micro BCA protein assay kit (Thermo Fisher Scientific), with the BSA supplied as standard. Cytokine concentrations in the extracts were measured on multiplex panels using MESO QuickPlex SQ 120 (MSD). The following cytokines were measured: IL-1α, IL-1RA, IL-8, IL-18, IL-33, IFN-γ, IL-22, IP10/CXCL-10), macrophage inflammatory protein 3α (MIP3α), MDC, TARC/CCL17, and TSLP. The analysis of NMF was performed according to a method previously described in detail elsewhere (90), with slight modifications. Briefly, NMF components (histidine, 2-pyrrolidinone-5-carboxylic, and *trans*- and *cis*-urocanic acid) on the TS (number 5) were extracted with 600 μL of ultra-clean water and subsequently analyzed by high-performance liquid chromatography. The concentration of NMF components was normalized by protein amount, determined by the Pierce Micro BCA Protein Assay Kit (Thermo Fisher Scientific).

### Statistics.

We adopted a Bayesian multinomial logistic model, fit via Markov chain Monte Carlo (MCMC) using latent variables, as previously described (91). We included parameter-inclusion indicators as also described (91), allowing us to perform Bayesian variable selection. The MCMC algorithm was run until we had 5,000 draws from the joint posterior distribution to derive Monte Carlo estimates of the parameters. The algorithm was run 6 times with different starting values to ensure it had converged. Predictive accuracy was assessed using the empirical postpredictive distribution. Due to the limited size of the data set, this was not split into training/validation/test sets, but rather training and accuracy assessments were based on the same data. This can tell us whether the model fits the data well, but not if it generalizes to unseen data. We also fit a version of the model that was adjusted for immunosuppression to determine whether the effects of systemic immunosuppression for management of AD could explain the differences identified between patient cohorts. This was done by adding an immunosuppression indicator (1 if patient is on immunosuppression, 0 otherwise) into the model as another covariate. Its inclusion in the model was guaranteed, and not a part of the variable selection scheme.

Box-and-whisker plots were constructed using ggplot2 default settings. The box width corresponds to the interquartile range (IQR), i.e., the distance between the 25th percentile and the 75th percentile. The vertical bar within each box represents the median, or 50th percentile. The whiskers extend as far as the largest or smallest value (direction-dependent) that is at most 1.5 times the IQR from the box. Outliers are any values that go beyond this 1.5 × IQR threshold.

### Study approval.

The study was conducted in accordance with the Declaration of Helsinki and was approved by the Research Ethics Committee of Children’s Health Ireland at Crumlin, Dublin, Ireland. Written informed consent from parents/guardians and patient assent (where feasible) was obtained prior to study enrolment.

### Data availability.

The data that support the findings of this study are available from the corresponding author upon request. Values for all data points in graphs are reported in the Supporting Data Values file. A link to the statistical code used is provided in Supplemental Data Set 6.

## Author contributions

RMM, JC, and ADI designed the study and acquired funding to complete it. JC recruited study participants and collected biological samples. JC and TJC processed and analyzed blood samples. JAG, AMT, MBT, and MS analyzed microbiological samples. IJ and SK analyzed stratum corneum tape strips. DJD performed the statistical analysis, which was supervised by AW. JC, DJD, and RMM wrote the manuscript. ADI, JAG, IJ, SK, TJC, AMT, MBT, MS, and AW edited the manuscript.

## Supplementary Material

Supplemental data

Supporting data values

## Figures and Tables

**Table 1:**
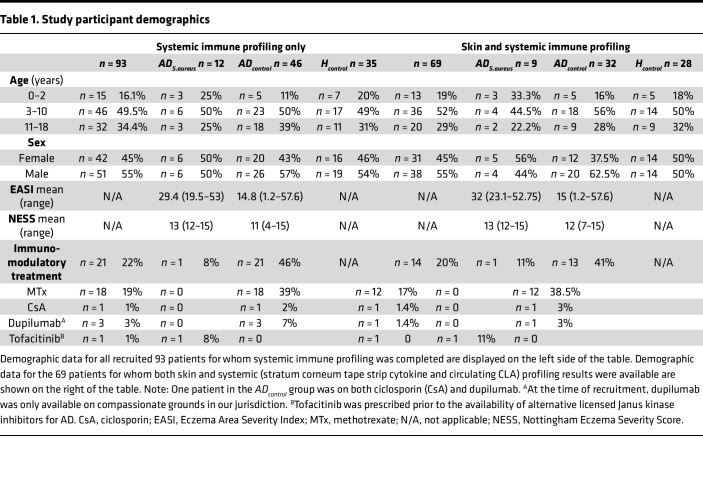
Study participant demographics

**Table 2:**
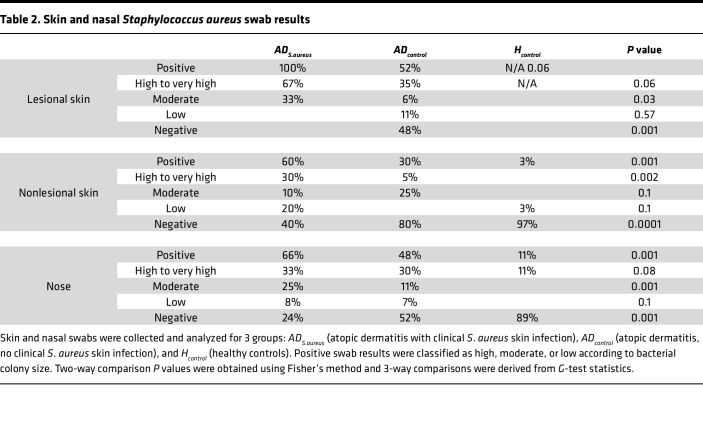
Skin and nasal *Staphylococcus* *aureus* swab results

**Table 3:**
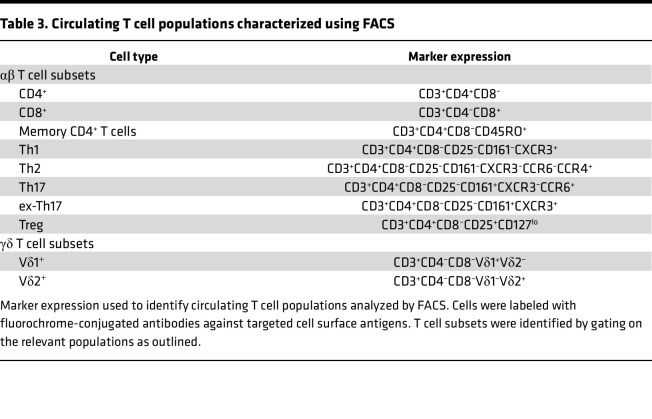
Circulating T cell populations characterized using FACS

**Table 4:**
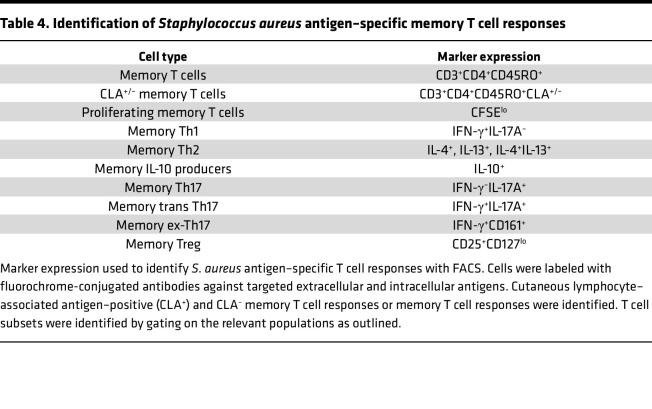
Identification of *Staphylococcus* *aureus* antigen–specific memory T cell responses

## Version 1 05/08/2024

Electronic publication
