# Supplementary material for: Distinct T cell signatures are associated with Staphylococcus aureus skin infection in pediatric atopic dermatitis
Source: JCI Insight. 2024 May 8;9(9):e178789. doi: 10.1172/jci.insight.178789 (PMC11141913; doi:10.1172/jci.insight.178789)
Supplement: Supplemental data [file jciinsight-9-178789-s136.pdf]

Supplemental Figure 1:

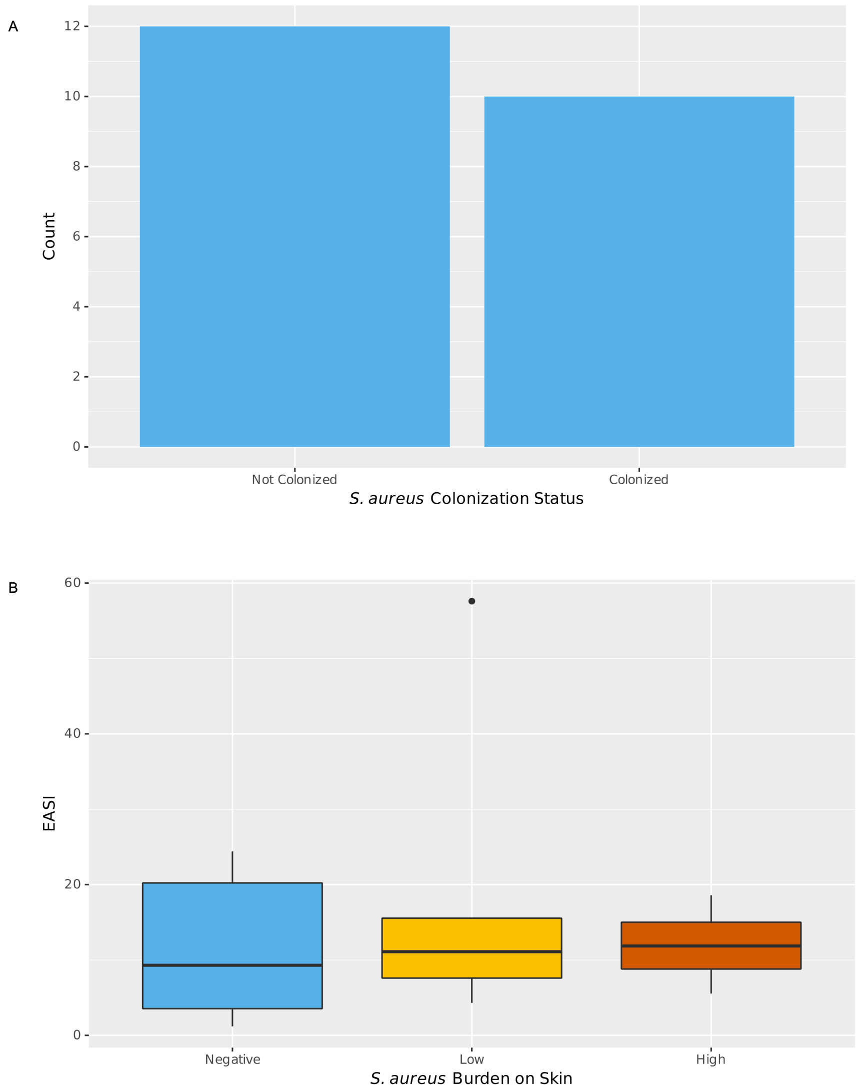

***S. aureus* colonization status of AD patients on systemic immunomodulatory treatment and burden of *S. aureus* skin colonization in $AD_{control}$ patients on systemic immunomodulatory therapy**

Subjects on systemic immunomodulatory treatment for management of AD (n = 22) in the total study cohort were grouped according to *S.aureus* skin colonization status (A). The number of patients is indicated on the x-axis and colonization status is shown on the y-axis. There was no significant correlation between colonization status systemic immunomodulatory treatment as assessed using the chi-squared test. (p value = 0.08). Colonized and on systemic immunomodulation: n = 10 . Not colonized and on systemic immunomodulation: n = 12

Subjects on systemic immunomodulatory therapy within the $AD_{control}$ cohort (n = 21) were grouped according to their burden of *S.aureus* skin colonization: negative (n=12), low (n=5) and high (n=4), to determine if thosewith high colonization represented therapeutic non-responders (B). The burden of *S. aureus* colonization is indicated on the x-axis and EASI score is indicated on the y-axis. There was no correlation between EASI score and the burden of *S.aureus* skin colonization using the t-test of Spearman correlation in $AD_{control}$ subjects on systemic immunomodulatory therapy (p value = 0.63).

Supplemental Figure 2: Gating for circulating T cell populations

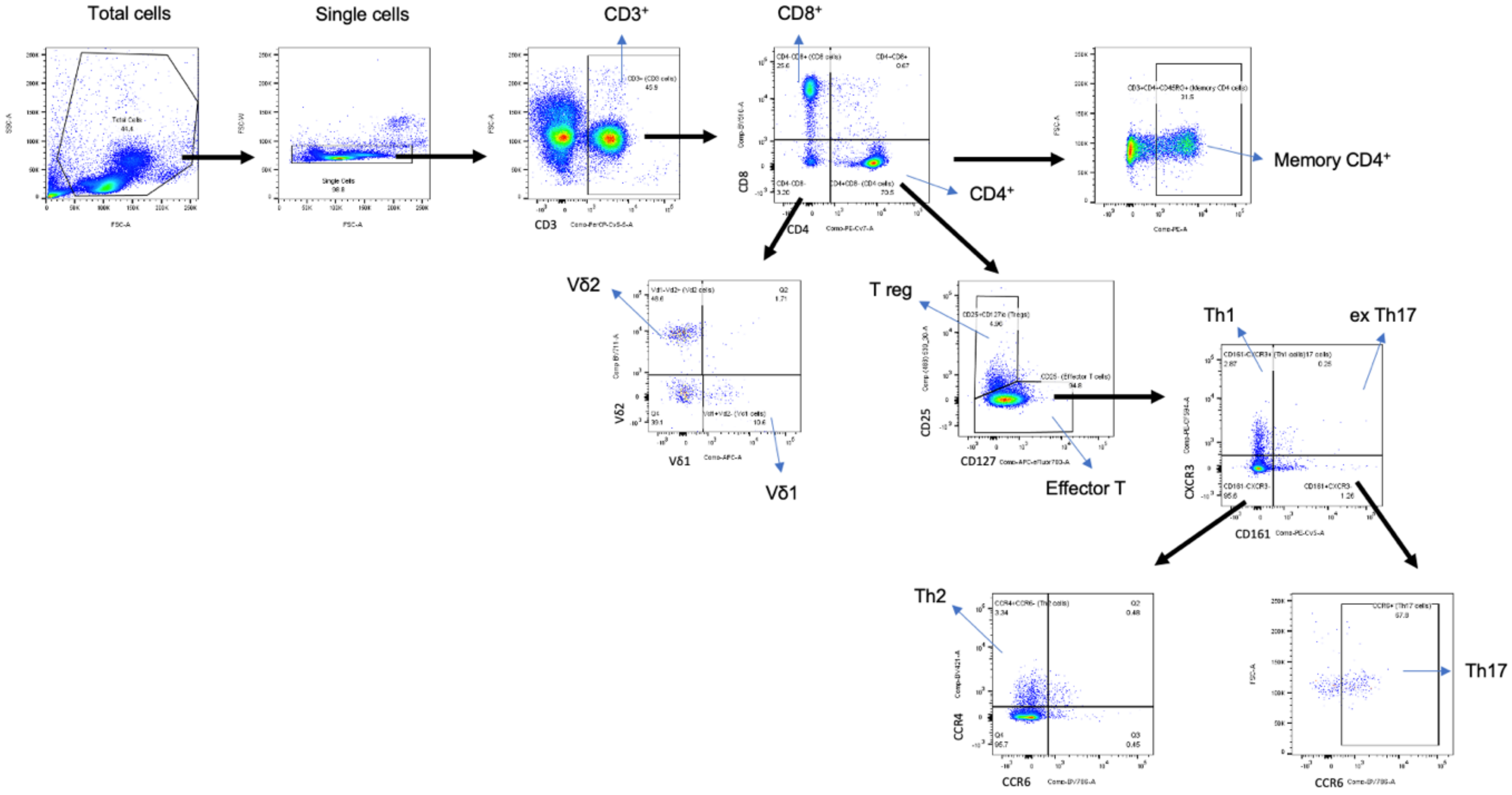

Supplemental Figure 3: Gating for CLA$^{+/-}$ *S. aureus* antigen-specific memory responses

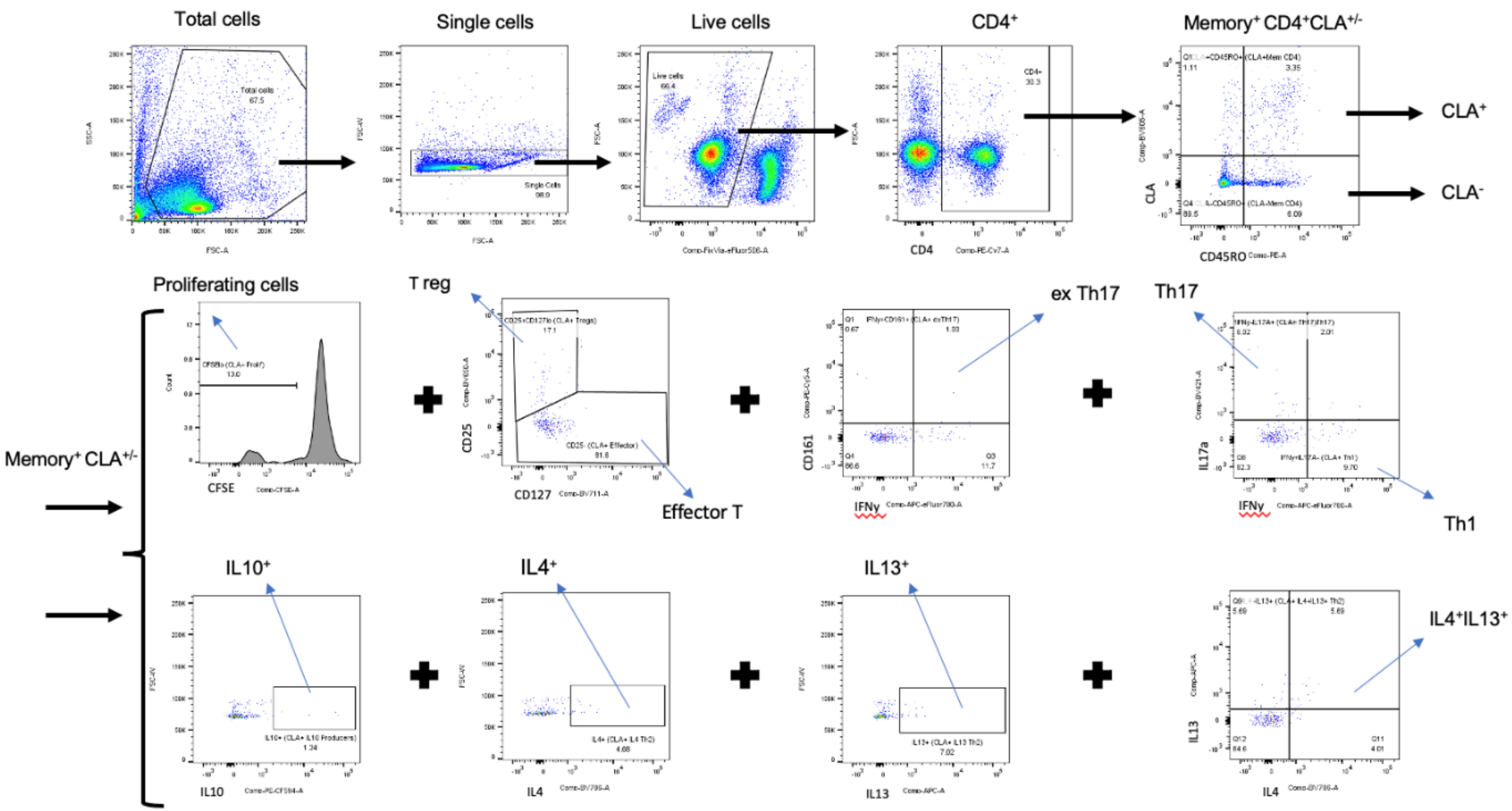

Supplemental Figure 4: Gating for total *S. aureus* antigen-specific memory responses

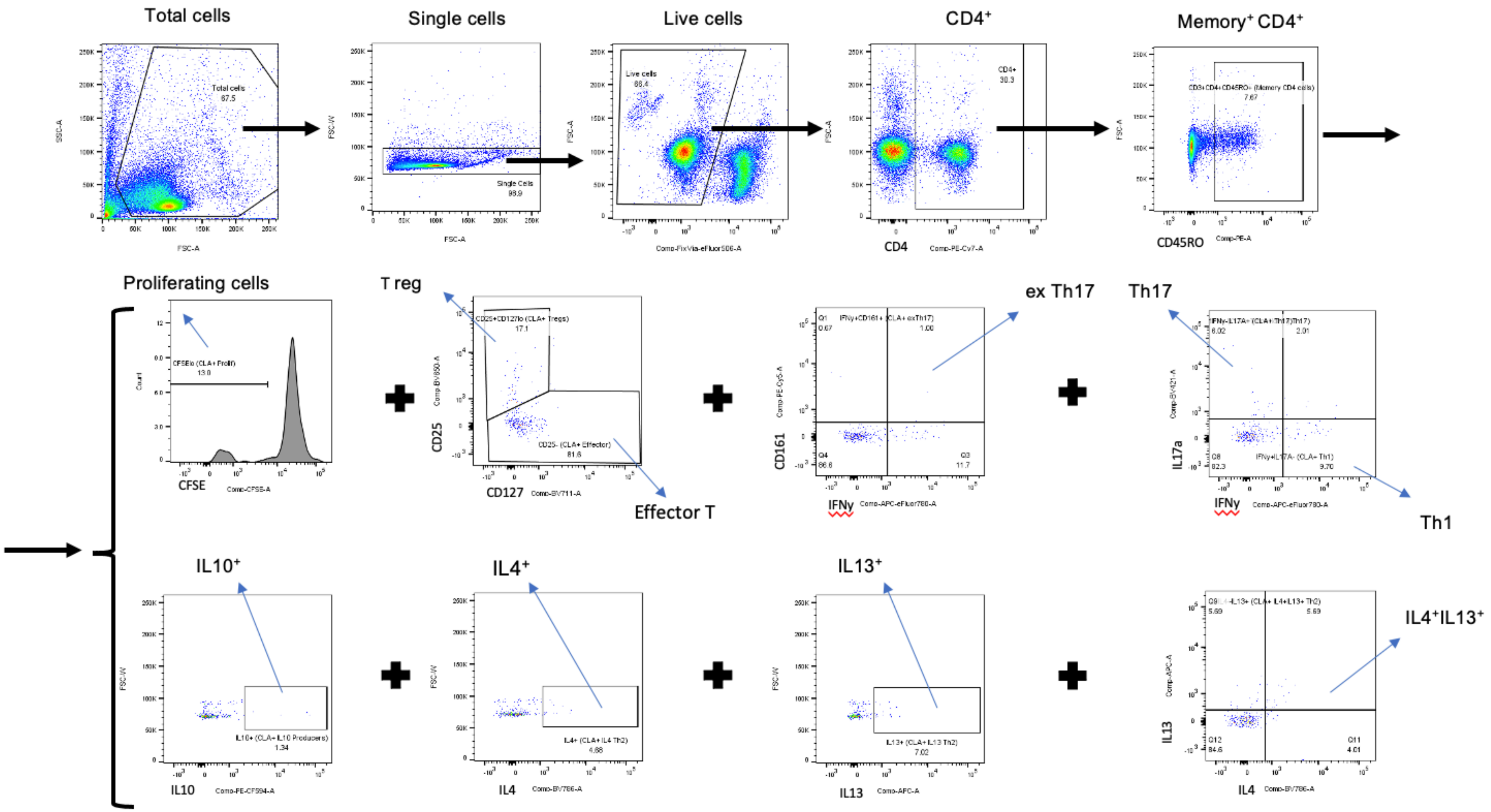

Supplemental Data 5: MIATA / MIANKA Checklist

#	Required	If available	Optional	MIANKA & MIATA Sub-Modules
				Module 1 - Sample
				**Module 1A - Donor**
1.1	✔			Essential donor info
				**Module 1B Source**
1.2	✔			Source of cell material
1.3	✔			Collection methodology
1.4		✔		anti-coagulant, if available
1.5		✔		Transportation/storage conditions for unprocessed samples, if available
1.6	✔			Cell processing methodology
1.7		☐		Median time and ranges from sample collection until end of cell processing, if available
1.8		☐		Cut-offs, if used
				**Module 1C - Cryopreservation and Storage**
1.9	✔			Fresh or cryopreserved
				If cryopreserved
1.10	☐			devices used
1.11	☐			freezing process
1.12	☐			medium used for freezing
1.13		☐		Median time and temperature for each transportation and storage step, if available
1.14		☐		Cut-offs, if used
				**Module 1D - Cell Counting**
1.15	✔			Median cell yield and viability (where available)
1.16		☐		before freezing
1.17		☐		after thawing
1.18		☐		after overnight resting
1.19		☐		Cut-offs, if used
1.20	✔			Cell counting methodology
1.21			☐	*Optional: Additional assessments*
				Module 2 - Assay
				**Module 2A - Medium/serum**
2.1	✔			Medium/(serum) details
2.2	✔			Pretesting info
				**Module 2B - Assay**
2.3		☐		Treatment procedures of cells prior to assay, if applicable
2.4	✔			Sufficient assay details
				**Module 2C - Controls**
2.5	✔			Internal assay controls
2.6		☐		Acceptance criteria, if available
2.7		☐		External reference samples, if used
2.8		☐		Assay acceptance criteria, if available
				Module 3 - Data Acquisition
				**Module 3A - Equipment and software**
3.1	✔			Equipment and software version
3.2		☐		Basic equipment settings, if available
				**Module 3B - Acquisition Strategy and Gating**
3.3	✔			Detailed gating strategy or strategy for establishing spot detection parameters
3.4	✔			Representative data set
3.5		☐		Mean,median, ranges of event counts for relevant populations, if available
3.6			☐	*Optional: Unusual strategies explained*
3.7			☐	*Optional: Review of raw data*
				Module 4 - Results
				**Module 4A - Raw data**
4.1		☐		Background and ag-specific reactivity levels, if available
4.2		☐		Cut-off specifications and # of tests OOS, if available
4.3	✔			Accessibility of raw data addressed?
				**Module 4B - Response determination**
4.4	✔			Definition of positive reactivity (above background) including tests applied
4.5		☐		Parameters, software and version used for response determination, if applicable
4.6	✔			Response definition predefined or post-hoc?
4.7		☐		Definition of response induced by treatment, if applicable
4.8		☐		Any data excluded and why, if applicable?
4.9			☐	*Optional: Why test was used*
				Module 5
				**Module 5A - General Lab Operation**
5.1	✔			Guidance of lab operations
5.2		✔		Laboratory accreditions and certifications, if available
5.3			☐	*Optional: Details on audits*
				**Module 5B - Standardization**
5.4	✔			Status of protocols
				**Module 5C - Qualification/Validation**
5.5	✔			Status of assays
5.6			☐	*Optional: Specific performance criteria*

Supplemental Data 6: Link to statistical coding

https://github.com/DanDempsey/AD_Study_Code
